# Zyxin stabilizes RIG-I and MAVS interactions and promotes type I interferon response

**DOI:** 10.1038/s41598-017-12224-7

**Published:** 2017-09-19

**Authors:** Takahisa Kouwaki, Masaaki Okamoto, Hirotake Tsukamoto, Yoshimi Fukushima, Misako Matsumoto, Tsukasa Seya, Hiroyuki Oshiumi

**Affiliations:** 1 Department of Immunology, Graduate School of Medical Sciences, Faculty of Life Sciences, Kumamoto University, 1-1-1, Honjo, Chuo-ku, Kumamoto 860-8556 Japan; 2 Department of Microbiology and Immunology, Graduate School of Medicine, Hokkaido University, Kita-Ku, Sapporo 060-8556 Japan; 3 JST, PRESTO, 1-1-1- Honjo, Chuo-ku, Kumamoto 060-8556 Japan

## Abstract

RIG-I and MDA5 are cytoplasmic viral RNA sensors that belong to the RIG-I-like receptors (RLRs), which induce antiviral innate immune responses, including the production of type I interferon and other pro-inflammatory cytokines. After recognition of viral RNA, the N-terminal caspase activation and recruitment domains (CARDs) of RIG-I and MDA5 bind to a CARD in the MAVS adaptor molecule, resulting in MAVS oligomerization and downstream signaling. To reveal the molecular mechanism of MAVS-dependent signaling, we performed a yeast two-hybrid screening and identified zyxin as a protein that binds to MAVS. Zyxin co-immunoprecipitated with MAVS in human cells. A proximity ligation assay showed that zyxin and MAVS partly co-localized on mitochondria. Ectopic expression of zyxin augmented MAVS-mediated IFN-β promoter activation, and knockdown of zyxin (*ZYX*) attenuated the IFN-β promoter activation. Moreover, *ZYX* knockdown reduced the expression of type I IFN and an interferon-inducible gene after stimulation with polyI:C or influenza A virus RNA. Interestingly, physical interactions between RLRs and MAVS were abrogated by *ZYX* knockdown. These observations indicate that zyxin serves as a scaffold for the interactions between RLRs and MAVS.

## Introduction

The innate immune system is essential for controlling viral infection. Retinoic acid-inducible gene I (RIG-I)-like receptors (RLRs), including RIG-I and myeloma differentiation-associated protein 5 (MDA5), recognize cytoplasmic double-stranded RNA (dsRNA) and trigger the signal to induce type I interferon (IFN) expression via mitochondrial antiviral signaling protein (MAVS)^1–3^. The RIG-I and MDA5 proteins contain two caspase activation and recruitment domains (CARDs), a helicase domain, and a C-terminal domain (CTD). N-terminal CARDs are essential for triggering the signal, and the helicase domain and CTD are responsible for dsRNA binding^4^. The RIG-I CTD also plays a regulatory role in RIG-I activation. In resting cells, the CTD suppresses the CARD activation^5^. Upon recognition of viral dsRNA, RIG-I assembles along the dsRNA, and the suppression by CTD is released^6^. RIG-I–dsRNA nucleoprotein filament formation leads to the assembly of RIG-I N-terminal CARDs, resulting in the formation of a 2CARD tetramer structure^7^. A ubiquitin ligase, Riplet, mediates K63-linked polyubiquitination of RIG-I, which promotes the access of another ubiquitin ligase, TRIM25, to the RIG-I CARDs; the TRIM25-mediated K63-linked polyubiquitin chain stabilizes the 2CARD tetramer structure^8–10^. Although the MDA5 CTD does not play a suppressive role in 2CARD activation, MDA5 in resting cells is suppressed by phosphorylation mediated by RIOK3 and other unidentified protein kinases, and phosphatase 1 plays a crucial role in MDA5 activation^11–13^. MDA5 also assembles along dsRNA, leading to the formation of the 2CARD tetramer structure of MDA5^7,14^.

RIG-I–dsRNA nucleoprotein filaments are recruited to mitochondria, where MAVS localizes, by 14-3-3ε^15^. The 2CARD tetramer of RIG-I or MDA5 acts as a core for initiating MAVS protein polymerization, leading to MAVS prion-like fiber formation^7,16^. Prion-like aggregation of MAVS on the outer membranes of mitochondria leads to activation of several downstream factors, such as TBK1, IKK-ε, and TRAF proteins^1^. TBK1 is a protein kinase, and its autophosphorylation at serine 172 is essential for TBK-1-mediated IRF-3 activation^17,18^. The downstream factors activate transcription factors, including IRF-3 and NF-κB required for type I IFN and other pro-inflammatory cytokine expression. There are several accessory factors involved in this MAVS-dependent signaling pathway^19^. DDX3 is a cytoplasmic RNA helicase. Schroder M *et al*. first reported that DDX3 is involved in RIG-I-mediated type I IFN production^18^. Later, we reported that DDX3 binds to RIG-I and MAVS, and facilitates RIG-I-RNA binding, resulting in enhanced type I IFN production^20^.

Zyxin was first identified as a protein localized at adhesion plaques and the ends of actin filaments^21^. The protein comprises 3 LIM domains in its C-terminal region. The LIM domain is known to mediate protein–protein interactions. Previous studies have revealed that the zyxin is involved in focal adhesion and is required for actin polymerization^22^. Later studies reported that zyxin localizes not only at adhesion plaque but also spindle poles during mitosis^23^. However, zyxin-deficient mice grow and breed normally^24^. Zyxin has pleiotropic functions and is known to be involved in other intracellular events, such as Hippo signaling, which controls cell growth and tissue homeostasis^25^. In the Hippo pathway, zyxin serves as a scaffold protein that stabilizes the interaction of Lats2 and Siah2, which results in Lats2 ubiquitination and degradation, leading to cancer cell migration and proliferation^26^. However, the roles of zyxin in antiviral innate immune responses are largely unknown. Here, we identified zyxin as a protein that binds to MAVS. Zyxin were partly co-localized with MAVS at mitochondria and bound to MAVS in the presence and absence of stimulation. Zyxin also bound to RIG-I and MDA5, and knockdown of zyxin abrogated the interaction between RLRs and MAVs, thereby reducing MAVS-mediated signaling. Our data indicate that zyxin serves as a scaffold for the interactions between RLRs and MAVS.

## Results

### Identification of zyxin as a protein that binds MAVS

**Figure 1:**
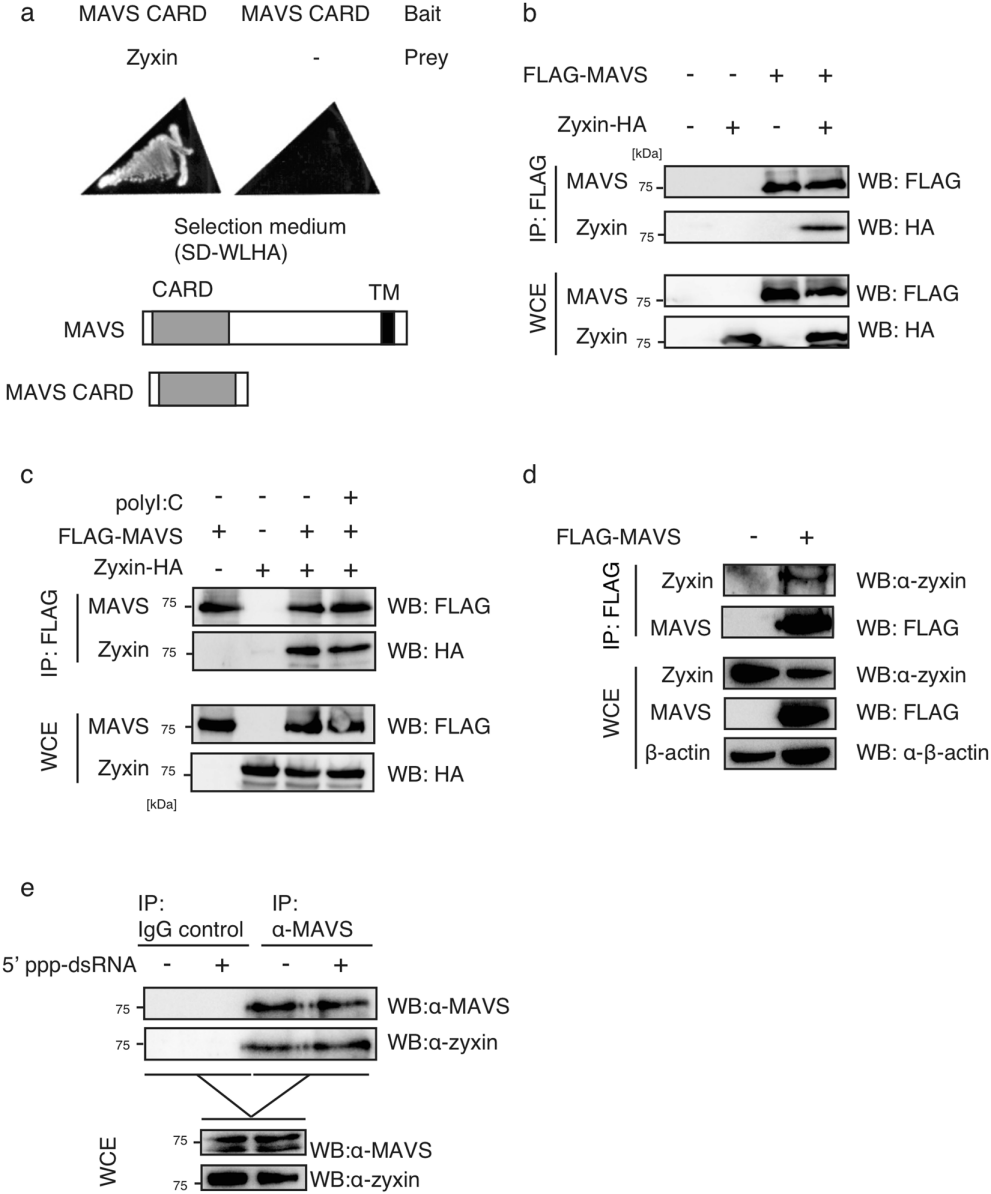
Zyxin binds to MAVS. (**a**) A plasmid carrying the Gal4 DNA-binding domain fused to a MAVS CARD fragment (Bait) was transformed into AH109 yeast cells either with a plasmid carrying the Gal4 activation domain fused to zyxin, or with empty vector (Prey). The transformed cells were inoculated on to selective medium (SD-WLHA). The yeast cells grew on the selective plates when the 2 proteins interacted with each other. (**b**) FLAG-MAVS and/or Zyxin-HA vectors were transfected into HEK293FT cells for 24 h, then cells were lysed with lysis buffer (whole cell extract, WCE). The proteins were immunoprecipitated with an anti-FLAG antibody and subjected to SDS-PAGE. The proteins were detected by western blotting (WB) with the indicated antibodies (against HA and FLAG). (**c**) HEK293FT cells expressing FLG-MAVS and Zyxin-HA were mock-transfected or transfected with polyI:C for 6 h, and whole cell extract (WCE) was subsequently prepared. WCE were used for immunoprecipitation with an anti-FLAG antibody, and immunoprecipitates were subjected to SDS-PAGE. The proteins were detected by WB with the indicated antibodies. (**d**) FLAG-MAVS or empty vector was transfected into HEK293FT cells for 24 h. Proteins from WCEs were immunoprecipitated with anti-FLAG antibody. The proteins were subjected to SDS-PAGE. Endogenous zyxin was detected by WB with anti-zyxin antibodies, and other proteins were detected with the indicated antibodies. (**e**) HEK293FT cells were mock-transfected or transfected with 5′-pppRNA for 6 h. Proteins in WCEs of HEK293FT cells were immunoprecipitated with anti-MAVS antibody or control IgG. Immunoprecipitates were subjected to SDS-PAGE, and endogenous proteins were detected with anti-MAVS and anti-zyxin antibodies. The original full blot images for western blotting can be found in Supplemental Fig. 2.

To isolate MAVS binding partners, bait plasmids carrying MAVS CARD were transformed into AH109 yeast cells together with prey plasmids carrying a human lung cDNA library, and then we performed a yeast two-hybrid screening using yeast selection plates. We obtained several candidates, including DDX3 as reported previously^20^. DDX3 has been shown to bind MAVS and RIG-I, and to promote RIG-I-mediated type I IFN expression^20^. Other groups also elucidated the requirement of DDX3 in MAVS-dependent type I IFN production^17,18^. In the screening, we identified another candidate, which encoded the C-terminal region of zyxin. We confirmed that AH109 yeast cells transformed with both a bait plasmid carrying MAVS CARD and a pray plasmid carrying zyxin C-terminal region could grow on yeast selection medium (SD-WLH) plates (Fig. 1a left panel), whereas yeast cells carrying the bait plasmids and empty prey plasmids failed to grow on the selection plate (Fig. 1a right panel), suggesting the interaction between zyxin and MAVS CARD in yeast cells. The role of zyxin in the regulation of MAVS-dependent signaling pathway is largely unknown; thus, we focused on the function of zyxin in the innate immune antiviral response.

First, we examined the physical interactions between MAVS and zyxin in human cells. We ectopically expressed HA-tagged zyxin (Zyxin-HA) in HEK293FT cells together with FLAG-tagged MAVS (FLAG-MAVS), and performed an immunoprecipitation assay with an anti-FLAG antibody. Zyxin-HA co-immunoprecipitated with FLAG-MAVS (Fig. 1b). Short and long polyI:C are recognized by RIG-I and MDA5, leading to MAVS-mediated type I IFN production^27^. Thus, we investigated if stimulation of cells by polyI:C transfection affected the physical binding between Zyxin-HA and FLAG-MAVS. Zyxin-HA co-immunoprecipitated with FLAG-MAVS in the presence and absence of stimulation with polyI:C (transfection) in our experimental conditions (Fig. 1c). Next, we investigated the interaction between FLAG-MAVS and endogenous zyxin. We ectopically expressed FLAG-MAVS in HEK293FT cells, and performed an immunoprecipitation assay with anti-FLAG antibody. Interestingly, endogenous zyxin co-immunoprecipitated with FLAG-MAVS (Fig. 1d). Moreover, endogenous zyxin co-immunoprecipitated with endogenous MAVS in the presence or absence of stimulation with a ligand for RIG-I, 5′-triphosphate RNA (5′ ppp-RNA) (Fig. 1e). These data suggest that the zyxin constitutively binds to MAVS.

**Figure 2:**
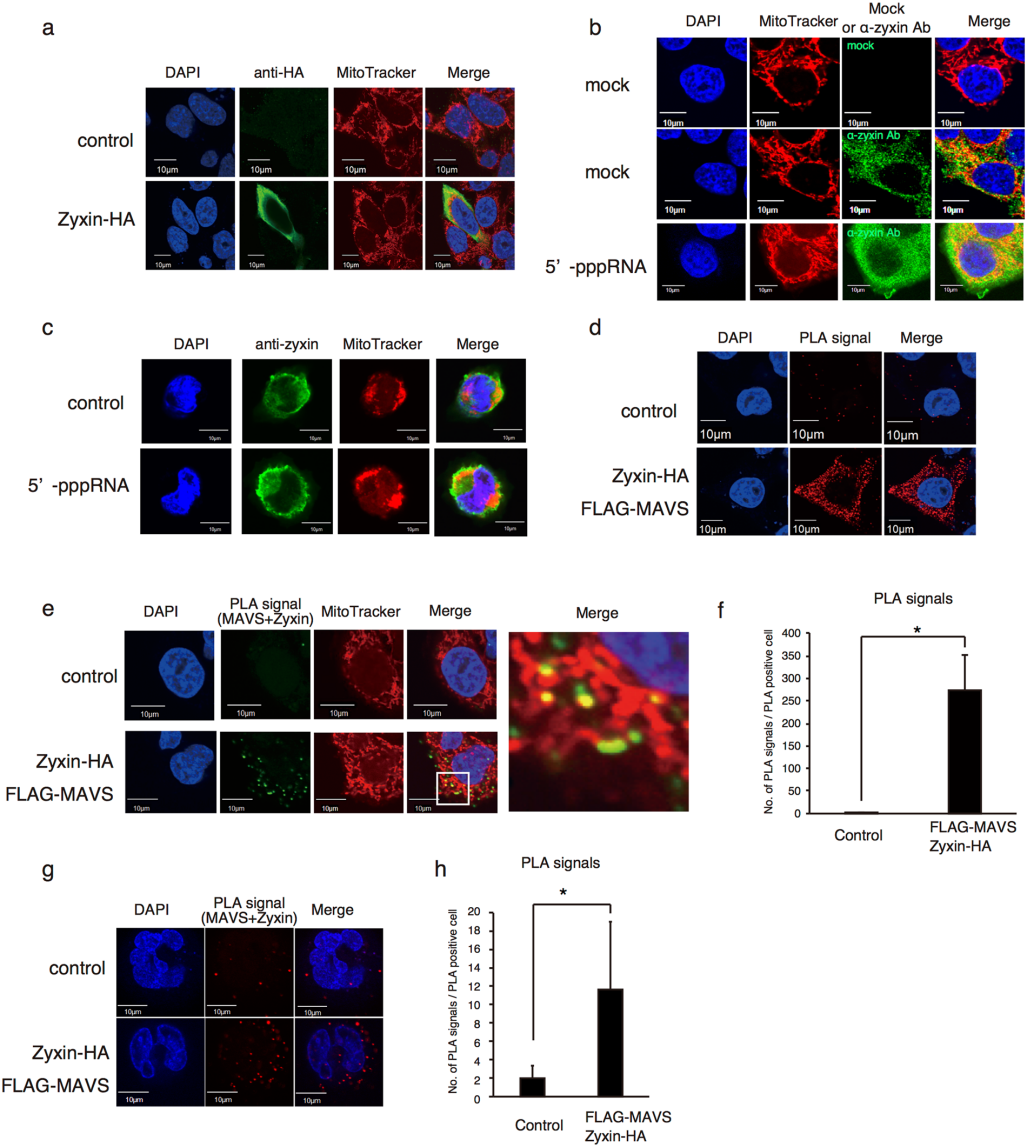
Subcellular localization of zyxin. (**a**) Empty vector or Zyxin-HA expressing vector was transfected into HeLa cells for 24 h. Cells were labeled with anti-HA polyclonal rabbit antibody and were stained with anti-rabbit Alexa 488 antibodies, DAPI, and MitoTracker Red. Cells were observed by confocal microscopy. (**b**) HeLa cells were mock-transfected or transfected with 5′ppp-RNA for 6 h. Cells were then labeled with control or anti-zyxin antibody and stained with anti-rabbit Alexa 488 antibody, DAPI, and MitoTracker Red. Cells were observed by confocal microscopy. (**c**) THP-1 macrophages were mock-transfected or transfected with 5′-pppRNA transfection for 6 h. Cells were labeled with control or anti-zyxin antibody and were stained with anti-rabbit Alexa 488 antibody, DAPI, and Mitotracker Red. Localization of zyxin was observed by confocal microscopy. (**d**) Empty (control), FLAG-MAVS, and Zyxin-HA vectors were transfected into HeLa cells for 24 h, then HeLa cells were fixed and labeled with anti-FLAG and anti-HA antibodies. A PLA was performed to detect the co-localization of zyxin and MAVS, and PLA signals are shown as red spots. (**e**–**h**) Empty (control), FLAG-MAVS and Zyxin-HA were expressed in HeLa (**e**,**f**) or THP-1 (**g**,**h**) cells. A PLA was performed to detect the co-localization of zyxin and MAVS, and PLA signals are shown in green. Cells were also stained with MitoTracker Red. Co-localization of PLA signals with mitochondria is shown in yellow. The numbers of PLA signals per PLA-positive HeLa cell (**f**) or THP-1 cell (**h**) were counted. The data represent mean ± standard deviation (SD). *P < 0.05 (t-test).

Next, we observed the subcellular localization of zyxin by immunofluorescence analysis via confocal microscopy and found that Zyxin-HA and endogenous zyxin was detected in the cytoplasm in HeLa cells in the presence and absence of stimulation with 5′ ppp-RNA (Fig. 2a,b). Cytoplasmic localization of zyxin was also detected in THP-1 cells (Fig. 2c). Because zyxin localization seemed to partially overlap the region where mitochondria localized, we performed a proximity ligation assay (PLA), in which PLA signals were detected if the 2 proteins were localized near each other^28,29^ in order to detect the co-localization of zyxin and MAVS. Zyxin-HA and FLAG-MAVS were transfected into HeLa cells, and the PLA was performed with anti-HA and anti-FLAG antibodies. We observed abundant PLA signals in HeLa cells expressing Zyxin-HA and FLAG-MAVS but not in control cells (Fig. 2d–f). Increased PLA signals was also detected in THP-1 cells expressing Zyxin-HA and FLAG-MAVS compared to control cells (Fig. 2g,h). To investigate if zyxin co-localized with MAVS on mitochondria, PLA signals and mitochondria were stained with fluorescein and MitoTracker Red, respectively. Interestingly, the PLA signals were partly localized to the mitochondria (Fig. 2e). The data indicate that zyxin binds to MAVS on the mitochondria outer membrane.

Although the expression of RIG-I and MDA5 is induced by polyI:C or type I IFN stimulation, MAVS expression is hardly altered by polyI:C stimuli (Fig. S1a). To investigate the expression pattern of zyxin, we stimulated human cells with polyI:C or IFN-α and determined the expressions of zyxin mRNA (*ZYX*) and protein. PolyI:C stimulation immediately induced IFN-β (*IFN-B1*) mRNA expression but hardly increased the *ZYX* mRNA expression in human cells, and stimulation with IFN-α markedly increased the expression of an IFN-inducible gene, IP-10 (*CXCL10*), but not *ZYX* (Fig. S1b–S1e). The endogenous zyxin protein level was also not influenced by polyI:C stimulation (Fig. S1f). Zyxin is known to be phosphorylated, so we investigated its phosphorylation before and after polyI:C stimulation using an anti-phospho-zyxin monoclonal antibody. Although polyI:C stimulation moderately reduced the level of phospho-zyxin, substantial amounts of phosphorylated zyxin were still detected at 12 h after polyI:C stimulation (Fig. S1f). These data suggest that zyxin is constitutively expressed and largely unaltered by stimulation with polyI:C or type I IFN.

### Zyxin promotes MAVS-mediated *IFNB1* promoter activation

**Figure 3:**
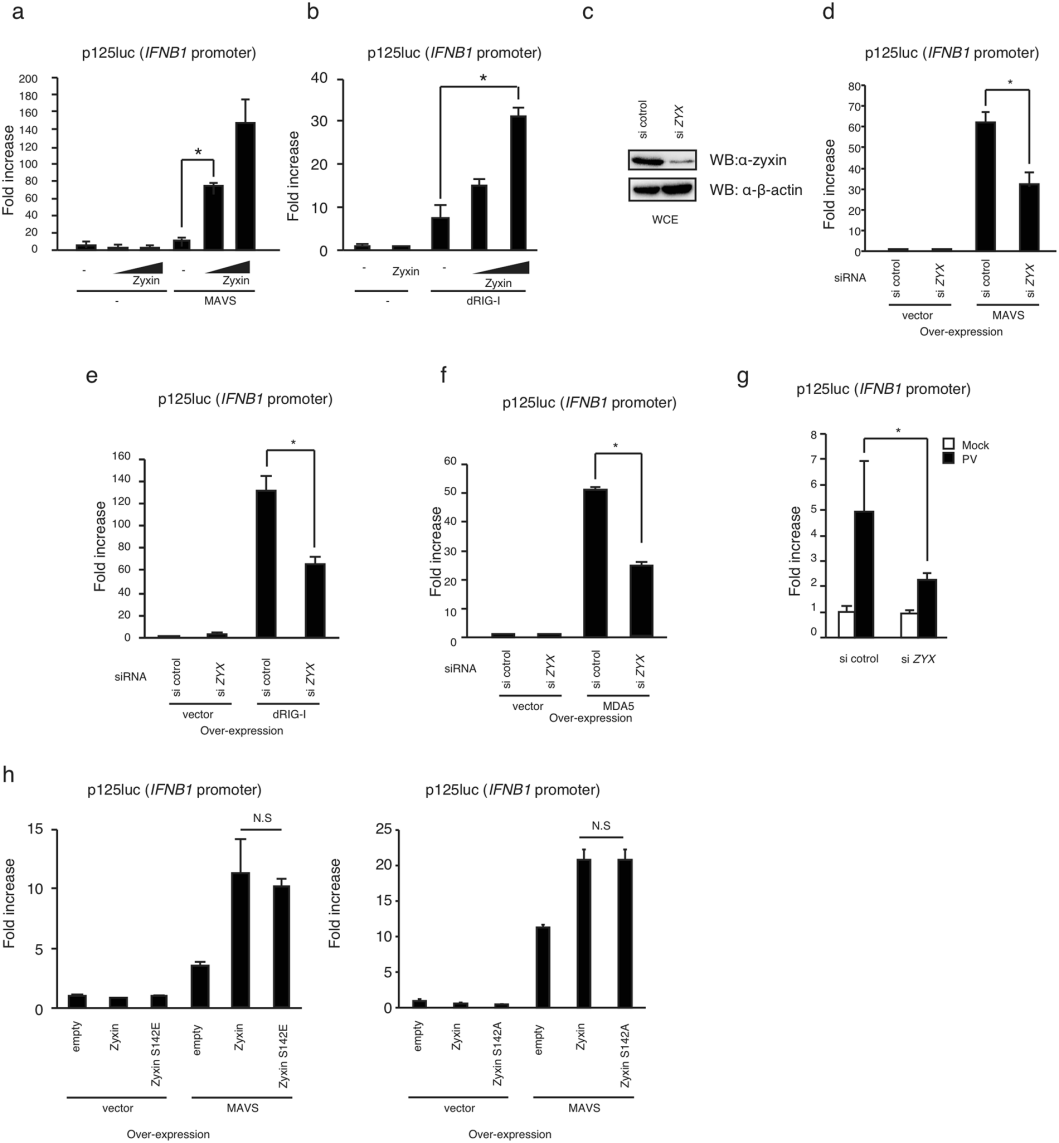
Zyxin promotes MAVS-mediated *IFNB1* promoter activation. (**a**,**b**) HEK293 cells were transfected with an empty vector or plasmids encoding MAVS (**a**), dRIG-I (**b**), and/or zyxin (0.1 μg, 0.3 μg) together with p125 luc (*IFNB1*) and Renilla luciferase vectors. Cells were lysed 24 h after transfection and reporter activities were determined by luciferase assay; luciferase activities were normalized to Renilla luciferase activity (internal control). Fold increase was calculated by dividing each value by the empty vector control. (**c**) siRNA for *ZYX* or negative control were transfected into HEK293 cells for 48 h. Cell lysates were prepared and were subjected to SDS-PAGE. Proteins were detected by western blotting with anti-zyxin and anti-β-actin antibodies. The original full blot images can be found in Supplemental Fig. 2. (**d**–**f**) siRNA for *ZYX* or negative control were transfected into HEK293 cells with MAVS (0.1 μg) (**d**), dRIG-I (0.1 μg) (**e**), or MDA5 (0.1 μg) (**f**) expression vectors and reporter plasmids (p125luc and Renilla luciferase) for 48 h. The fold increase was determined as described in (**a**). (**g**) Cells were transfected with siRNA for *ZYX* or negative control together with reporter plasmids (p125luc and Renilla luciferase). Two d after transfection, cells were infected with poliovirus (MOI = 1) for 24 h. Cells were lysed, and the fold increase was determined as described in (**a**). (**h**) HEK293 cells were transfected with MAVS, wild-type zyxin, a phospho-mimetic zyxin mutant (Zyxin-S142E), and phospho-deficient zyxin mutant (Zyxin-S142A) together with reporter plasmids (p125luc and Renilla luciferase). The fold increase was determined as described in (**a**). The data represent means ± SD, and the date are representative at least 2 independent experiments. *p < 0.05 (one-way ANOVA). “N.S.” represents not significant.

MAVS strongly induces the type I IFN expression, so we next investigated if zyxin is involved in MAVS-mediated signaling. For this purpose, we performed reporter gene assays using an *IFNB1* promoter reporter plasmid (p125luc). In resting cells, MAVS is not activated, and ectopic expression of MAVS leads to its autoactivation, resulting in the activation of the *IFNB1* promoter even in the absence of stimulation^20^. Unlike with MAVS, ectopic expression of zyxin alone failed to activate the *IFNB1* promoter in resting cells (Fig. 3a). In contrast, zyxin expression augmented the activation of the *IFNB1* promoter induced by ectopic MAVS expression (Fig. 3a). Overexpression of a RIG-I fragment, dRIG-I, which contains only the N-terminal 2CARD of the RIG-I protein and lacks the regulatory CTD, is known to activate MAVS, which leads to *IFNB1* promoter activation^2^. When MAVS was activated by dRIG-I overexpression, ectopic zyxin expression augmented dRIG-I-induced *IFNB1* promoter activation (Fig. 3b).

To further investigate the effects of zyxin on MAVS-mediated signaling, we performed knockdown assays. Short interfering (si)RNA for *ZYX* reduced endogenous zyxin protein levels (Fig. 3c) and significantly attenuated MAVS-mediated *IFNB1* promoter activation (Fig. 3d). In addition, siRNA for *ZYX* attenuated dRIG-I-induced *IFNB1* promoter activation (Fig. 3e). Like the dRIG-I fragment, ectopic MDA5 expression can activate MAVS-mediated signaling because the MDA5 protein does not contain a suppressor domain^5^. *ZYX* siRNA attenuated MDA5-mediated *IFNB1* promoter activation (Fig. 3f). MAVS is important for type I IFN expression after poliovirus infection^30^. We found that siRNA for *ZYX* moderately reduced *IFNB1* promoter activation after poliovirus infection (Fig. 3g). These data suggest that zyxin is a positive factor for the MAVS-dependent *IFNB1* promoter activation pathway. Although zyxin is a phosphoprotein, the Zyxin-S142E phosphomimetic mutation did not affect zyxin-mediated augmentation of *IFNB1* promoter activation (Fig. 3h), and the Zyxin-S142A phospho-deficient mutation, in which the phosphorylation site is replaced with Ala, also failed to affect the promoter activation (Fig. 3h), suggesting that the phosphorylation of zyxin is not a prerequisite for MAVS-mediated signaling.

**Figure 4:**
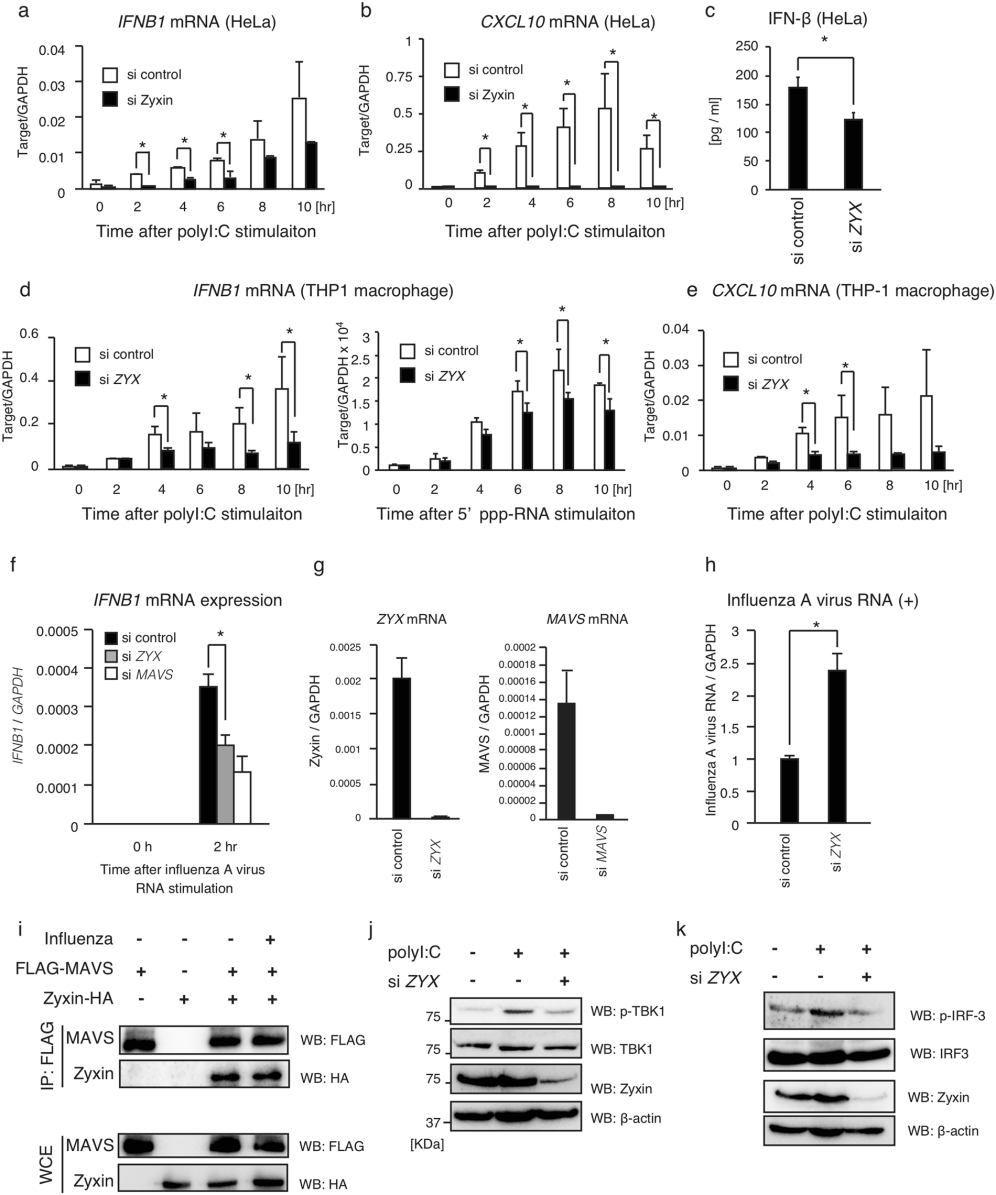
Zyxin is involved in a type I IFN expression pathway. (**a**,**b**) siRNA for *ZYX* or negative control were transfected into HeLa cells. Two d. after transfection, cells were transfected with polyI:C. Total RNA was extracted at the indicated time points. *IFNB1* and *CXCL10* mRNA expressions was determined by qPCR and normalized to *GAPDH*. The data represent means ± SD. They are representative of at least 2 independent experiments. *p < 0.05 (n = 3, t-test). (**c**) siRNA for *ZYX* or negative control was transfected into HeLa cells for 2 d. Cells were transfected with polyI:C for 24 h. IFN-β protein levels in cell culture medium were determined by ELISA. The data represent means ± SD. They are representative of 2 independent experiments. *p < 0.05 (n = 3, t-test). (**d**,**e**) siRNA for *ZYX* or negative control were transfected into THP-1 cells. Two d after transfection, cells were transfected with polyI:C or 5′ppp-RNA. Total RNA was extracted at the indicated time points. *IFNB1* and *CXCL10* mRNA expressions was determined by qPCR and normalized to *GAPDH*. The data represent means ± SD. They are representative of at least 2 independent experiments. *p < 0.05 (n = 3, t-test). (**f**,**g**) siRNA for negative control, *ZYX* and *MAVS* were transfected into HeLa cells for 2 d. Cells were transfected with *in vitro*-synthesized influenza A virus RNA. Total RNA was extracted at 0 and 2 h after transfection. The expression levels of *IFNB1* mRNA was determined by qPCR and normalized to *GAPDH* (**f**). Knockdown efficiency of *ZYX* in non-stimulated samples was determined by qPCR (**g**). *P < 0.05 (t-test). (**h**) siRNA for negative control and *ZYX* were transfected into HeLa cells for 2 d. Cells were infected with influenza A virus for 24 h, and total RNA was extracted. Reverse transcription was performed using a positive strand-specific primer. Cytoplasmic positive strand RNA levels were determined and normalized to *GAPDH*. The data represent means ± SD. They are representative of 2 independent experiments. *p < 0.05 (t-test). (**i**) HEK293FT cells transfected with FLAG-MAVS and Zyxin-HA were infected with influenza A virus at MOI = 10 for 24 h, and then cell lysate were prepared. Immunoprecipitation was performed with anti-FLAG antibody. Whole-cell extract and immunoprecipitates were subjected to SDS-PAGE, and the proteins were detected by western blotting. (**j**,**k**) HEK293 cells were transfected with siRNA for negative control or *ZYX* for 2 d. Cells were then stimulated with polyI:C for 6 h, and cell lysates were prepared. Cell lysates were subjected to SDS-PAGE, and the proteins were subsequently detected with the indicated antibodies. The original full blot images for western blotting can be found in Supplemental Fig. S2.

Next, we examined the effects of zyxin on endogenous *IFNB1* and *CXCL10* mRNA expression by MAVS. PolyI:C stimulation increased the expression of *IFNB1* and *CXCL10* mRNA, and siRNA for *ZYX* reduced the expression of those genes in HeLa cells (Fig. 4a,b). IFN-β protein production induced by polyI:C transfection was also reduced by *ZYX* knockdown (Fig. 4c). Knockdown of *ZYX* also reduced the expression of *IFNB1* and *CXCL10* mRNA in THP-1 macrophages in response to polyI:C or 5′ppp-RNA stimulation (Fig. 4d,e). Stimulation with influenza A viral RNA, which is a ligand for RIG-I^31^, induced the expression of *IFNB1*, and siRNA for *ZYX* reduced *IFNB1* mRNA expression as did siRNA for *MAVS* (Fig. 4f,g). Moreover, siRNA for *ZYX* increased viral replication in cells infected with the influenza A virus (Fig. 4h). We confirmed that Zyxin-HA bound FLAG-MAVS during influenza A virus infection (Fig. 4i). When MAVS is activated, TBK1 is phosphorylated, which is essential for TBK1-mediated IRF-3 phosphorylation^17^. TBK1 and IRF-3 phosphorylations were detected after stimulation with polyI:C, and that phosphorylations were reduced by *ZYX* knockdown (Fig. 4j,k). Taken together, these data indicate that zyxin is a positive factor in the MAVS-dependent type I IFN expression pathway.

### Zyxin stabilizes physical interaction between RLRs and MAVS

**Figure 5:**
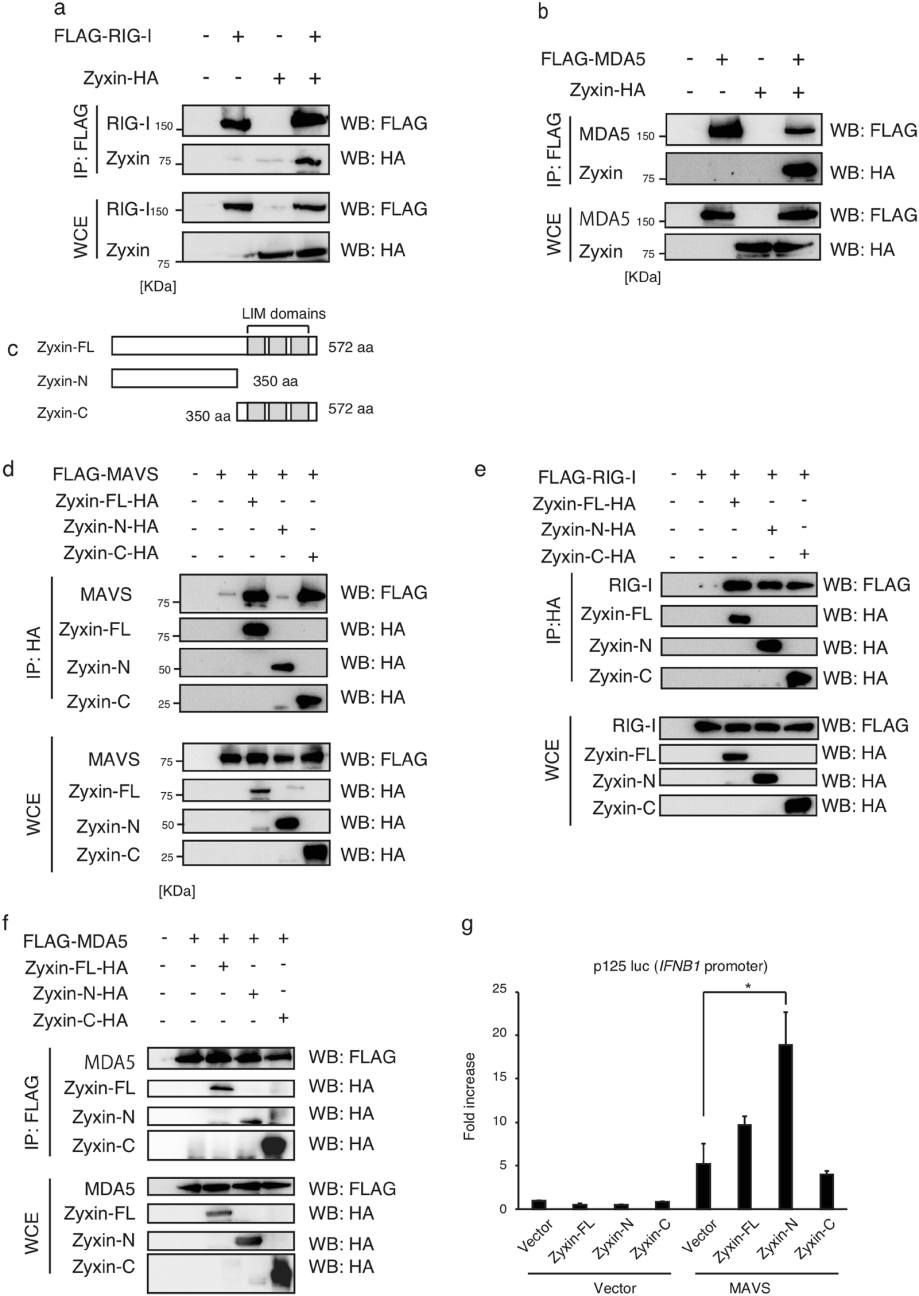
Zyxin binds to RLRs. (**a**,**b**) FLAG-RIG-I (**a**) or FLAG-MDA5 (**b**) expression vectors were transfected into HEK293FT cells together with Zyxin-HA for 24 h. The cell lysates were immunoprecipitated with anti-FLAG antibody and subjected to SDS-PAGE. Proteins were detected by WB with the indicated antibodies. (**c**) Schematic representation of zyxin partial fragments. Zyxin-FL, Zyxin-N, and Zyxin-C represent full-length, N-terminal, and C-terminal fragments, respectively. (**d**–**f**) Zyxin-HA fragments were transfected into HEK293FT cells together with FLAG-MAVS (**d**), FLAG-RIG-I (**e**), or FLAG-MDA5 (**f**) for 24 h. Cells were lysed, and immunoprecipitation was performed with anti-HA (**d**,**e**) or anti-FLAG (**f**) antibodies. WCEs and immunoprecipitates (IP) were subjected to SDS-PAGE, and the proteins were detected by WB using the indicated antibodies. (**g**) Zyxin-FL, Zyxin-N, and Zyxin-C encoding plasmids were transfected into HEK293 cells together with reporter plasmids (p125 luc reporter and Renilla luciferase). 24 h after transfection, the reporter gene assay was performed, and the activation of p125 luc reporter was determined. The data represent means ± SD. They are representative of 2 independent experiments. *P < 0.05 (one-way ANOVA). The original full blot images for western blotting can be found in Supplemental Fig. S3.

RIG-I and MDA5 directly bind to MAVS adaptor. Given that zyxin does not contain a catalytic domain, we expected that it might serve as a scaffold protein that stabilizes the interactions between RLRs and MAVS. To test this hypothesis, we investigated if zyxin could bind not only MAVS but also RLRs. Immunoprecipitation assays showed that Zyxin-HA co-immunoprecipitated with FLAG-tagged RIG-I (FLAG-RIG-I) and FLAG-tagged MDA5 (FLAG-MDA5) (Fig. 5a,b).

To determine the region responsible for the interactions, we constructed zyxin N-terminal fragment (Zyxin-N) and C-terminal fragment (Zyxin-C) (Fig. 5c). The Zyxin-C fragment contains 3 tandem LIM domains, which engaged in protein–protein interactions. An immunoprecipitation assay showed that Zyxin-C, but not -N, fragment co-immunoprecipitated with MAVS (Fig. 5d), whereas both fragments co-immunoprecipitated RIG-I and MDA5 (Fig. 5e,f). These data indicate that the zyxin C-terminal tandem LIM domains bind MAVS, and both N- and C-terminal fragments binds RLRs.

We next investigated the region of zyxin responsible for promoting MAVS-mediated signaling by reporter gene assay. Ectopic expression of Zyxin-N fragment exhibited strong ability to increase MAVS-mediated *IFNB1* promoter activation compared to full-length zyxin (Zyxin-FL) (Fig. 5g). In contrast, the Zyxin-C fragment alone failed to increase MAVS-signaling (Fig. 5g). These data suggest that the N-terminal region of zyxin is required for promoting MAVS-mediated signaling and that C-terminal region plays a regulatory role (see discussion).

**Figure 6:**
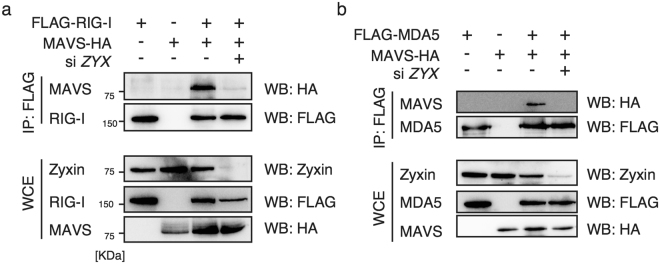
Zyxin stabilizes the interactions of MAVS and RLRs. (**a**,**b**) siRNA for negative control or *ZYX* were transfected into HEK293FT cells. After 48 h, cells were transfected with FLAG-RIG-I (**a**) or FLAG-MDA5 (**b**) together with a MAVS expression vector for 24 h. The cell lysates were immunoprecipitated with anti-FLAG antibody and subjected to SDS-PAGE. The proteins were detected by WB with the indicated antibodies. The original full blot images can be found in Supplemental Fig. S3.

**Figure 7:**
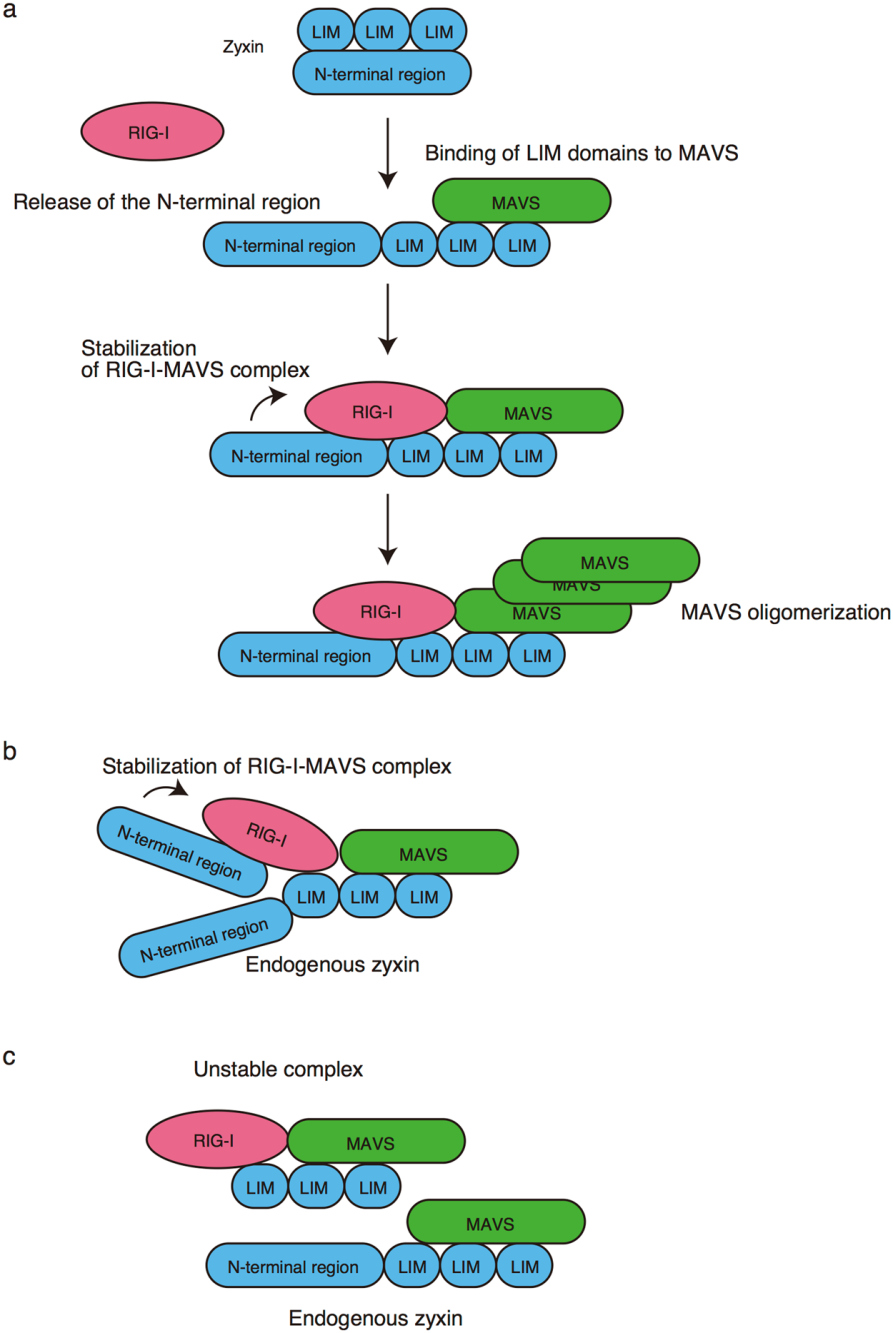
Model of zyxin-mediated stabilization of RIG-I-MAVS complex. (**a**) The C-terminal LIM domains of zyxin suppress the function of its N-terminal reigon. When the LIM domains bind to MAVS, the N-terminal region is released from its C-terminal region and bind to RIG-I, leading to stabilization of the RIG-I-MAVS complex required for MAVS oligomerization. (**b**) Ectopic zyxin-N expression can stabilizes the RIG-I-MAVS complex that includes endogenous zyxin. (**c**) Ectopic zyxin-C expression fails to stabilize the RIG-I-MAVS complex, because it lacks N-terminal region.

Next, we examined if knockdown of zyxin affects these interactions. We transfected siRNA for *ZYX* into HEK293FT cells expressing HA-tagged MAVS and FLAG-RIG-I, and performed immunoprecipitation with an anti-FLAG antibody. Interestingly, *ZYX* knockdown abrogated the physical interaction between RIG-I and MAVS (Fig. 6a). In addition, siRNA for *ZYX* abrogated MDA5-MAVS interactions (Fig. 6b). These data are consistent with our hypothesis that zyxin stabilizes the physical interaction between MAVS and RLRs (Fig. 7).

## Discussion

In this study, we focused on the function of zyxin in the MAVS-dependent signaling pathway. Zyxin was isolated in a two-hybrid screening, using MAVS as bait, and we confirmed the physical interaction between MAVS and zyxin by immunoprecipitation assays. Zyxin is constitutively expressed, and its expression is unaltered by stimulation with polyI:C or type I IFN. Knockdown of zyxin attenuated MAVS-mediated type I IFN expression in response to stimulation with RLR ligands, such as dsRNA and viral RNA. These data indicate that zyxin is a positive factor for MAVS-mediated signaling. In addition, we showed that knockdown of zyxin abrogated the interactions between RLRs and MAVS. These observations support the hypothesis that zyxin serves as a scaffold for the interaction between MAVS and RLRs.

RIG-I and MDA5 assemble along viral dsRNA^6,14^, leading to the formation of a 2CARD tetramer structure^7^. A K63-linked polyubiquitin chain stabilizes the RIG-I 2CARD tetramer structure^8^. The RIG-I-dsRNA complex is recruited to the mitochondria, where MAVS is localized, by 14-3-3ε^15^. The interaction of RIG-I with MAVS induces MAVS polymerization, leading to the formation of MAVS prion-like fiber. Previous studies have shown that the structure of the 2CARD tetramer of RIG-I acts as a core for MAVS polymerization^8^. Considering that the tandem 3 LIM domains of zyxin associate with both RIG-I and MAVS, zyxin is expected to serve as a scaffold for the interaction between the 2CARD tetramer core and MAVS, which promotes the formation of MAVS prion-like aggregation. It has been shown that zyxin assembles on the edges of other types of filaments. For instance, zyxin is included in focal adhesion complexes and is required for actin filament formation^22^. Another study reported that zyxin is localized at spindle poles during mitosis^23^. These observations imply a general role for zyxin in initiating protein polymerization and fiber formation. Considering that zyxin is involved in many cellular events, it is possible that zyxin deficiency could indirectly affect MAVS-dependent type I IFN expression. However, we found that endogenous zyxin co-immunoprecipitated with MAVS and that siRNA for *ZYX* abrogated interactions between MAVS and RLRs. These observations weaken the possibility that zyxin affect MAVS-dependent events in an indirect manner.

Deletion of the C-terminal region of zyxin augmented *IFNB1* promoter activation by ectopic expression of MAVS and zyxin. Therefore, it is expected that C-terminal region plays a suppressive role. This seems to be similar to that of RIG-I. RIG-I CTD is known to suppress its N-terminal CARDs in resting cells, and the binding of CTD to viral RNA release the RIG-I CARDs^5^. Ectopic expression of RIG-I CARD fragment activates MAVS via endogenous RIG-I^5^. It is possible that the binding of the C-terminal LIM domains to MAVS releases the N-terminal region of zyxin, which results in the stabilization of RIG-I-MAVS complex by the zyxin N-terminal region (Fig. 7). Further analysis is required to fully uncover the underlying mechanism.

There are several zyxin family proteins, including thyroid hormone receptor interactor 6 (TRIP6), Ajuba, and LIM domain-containing preferred translocation partner in lipoma (LPP)^32,33^. TRIP6 is a RIP2-associated protein that is involved in an NF-κB activation^34^. LIM domains of TRIP6 interact with various proteins, such as NF-κB p65 (RELA), c-FOS (FOS), and retinoid X receptors, and TRIP6 serves as a platform for the recruitment of signaling molecules^35,36^. Ajuba also serves as a scaffold and has pleiotropic functions. It is involved in an IL-1-mediated NF-κB activation pathway and a STAT1 signaling pathway^33^. Although previous *in vitro* studies have revealed the crucial role of zyxin in actin polymerization, zyxin-knockout mice do not have any developmental defects. Therefore, zyxin family members are expected to play redundant roles. In this study, we performed knockdown studies to evaluate the role of zyxin. *ZYX* siRNA markedly reduced *ZYX* mRNA levels. Although siRNA for *ZYX* reduced MAVS-mediated *IFNB1* mRNA expression, we still detected residual expression of *IFNB1*. This leaky phenotype might be caused by redundant roles among the zyxin family members. Considering that other zyxin-related proteins are involved in several signaling pathways, it is possible that zyxin is involved in not only MAVS-mediates signaling but also other signaling pathways, such as *CXCL10* expression pathway, because siRNA for ZYX exhibited more profound effects on the expression of *CXCL10* mRNA than that of *IFNB1* as shown in Fig. 4. MAVS is localized at both mitochondria and peroxisome, and peroxisomal MAVS induces antiviral gene expression but not interferon production^37^. Therefore, it is also possible that zyxin is involved in the signaling induced by not only mitochondrial MAVS but also peroxisomal MAVS.

Physical associations of zyxin with viral proteins have been reported. An accessory protein of HIV-1, nef, associates with zyxin^38^. The E6 protein of human papillomavirus type 6 also binds zyxin, which affects the subcellular localization of zyxin^39^. Zyxin deficiency has been shown to increase the mortality of mice during a bacterial infection, implying that zyxin is involved in an innate immune response pathway^40^, and RIG-I has been reported to sense bacterial nucleic acids^41^. However, the roles of zyxin in antiviral innate immune responses had not previously been elucidated. Our study provides new insights into the mechanisms of the antiviral innate immune response and elucidates a function of zyxin in antiviral innate immune response.

## Methods

### Cells and Reagents

HEK293 cells were cultured in D-MEM low glucose (Wako) with 10% FBS. HEK293FT, A549, and HeLa cells were culture in D-MEM high glucose (Wako) with 10% FBS. THP-1 cells were purchased from JCRB cell bank and cultured in RPMI-1640 (Wako) with 5% FBS. THP-1 cells were differentiated into macrophages with 60 ng/ml of PMA for 16 h. PolyI:C and 5′ pppRNA were purchased from GE Healthcare and Invivogen, respectively. Antibodies to the following proteins were purchased from the indicated manufacturers: anti-HA rabbit monoclonal antibody (SIGMA-Aldrich), anti-FLAG M2 mouse antibody (SIGMA-Aldrich), anti-β-actin mouse antibody (SIGMA-Aldrich), HRP-conjugated anti-rabbit donkey antibody (GE Healthcare), HRP-conjugated anti-mouse sheep antibody (GE Healthcare), anti-zyxin rabbit antibody (Cell signaling technology), anti-phospho-zyxin rabbit antibody (Cell signaling technology), anti-phospho TBK1 (Ser172) antibody (Cell signaling technology), anti-phospho-IRF-3 (Ser396) antibody (Cell signaling technology), anti-IRF-3 antibody (Cell signaling technology), anti-TBK1/NAK antibody (Abcam).

### Plasmids

Zyxin cDNA encoding the entire ORF was cloned into pCR-Blunt vector using primers, forward: 5′-ATG GCG GCC CCC CGC CCG TC-3′ and reverse: 5′-TCA GGT CTG GGC TCT AGC AG-3′ from human lung cDNA library. To make an expression plasmid, HA-tag was fused at the C-terminal end of the full length zyxin (pEF-BOS Zyxin-HA). pEF-BOS Zyxin-HA (S142E and S143E) was made by using primers, forward 1: 5′-CTC GAG GCC ACC ATG GCG GC-3′, reverse 1: 5′-AAA TCA ATT TCT TCC ACC TTC TCC CTG GGC TGT GGT GGG G-3′, forward 2: 5′-GAA GGT GGA AGA AAT TGA TTT GGA GAT CGA CTC TCT GTC C-3′ and reverse 2: 5′-GCG GCC GCC TAA GCG TAA TC-3′. pEF-BOS Zyxin-N-HA and pEF-BOS Zyxin-C-HA were made by using primers forward (N): 5′-CTA GAG ATC CCT CGA CCT CGA GGC CAC CAT GGC GGC-3′, reverse (N): 5′-GGT AAC TAG TGG ATC CGG GCC CTG GGG CCC CAG GGG- 3′, forward (C): 5′-ATC CCT CGA CCT CGA GGC CAC CAT GCT GAC TCT GAA GGA GGT GGA-3′, reverse (C): 5′-GGT AAC TAG TGG ATC CGG TCT GGG CTC TAG CAG TGT-3′.

### RNAi

Knockdown of *ZYX* was performed using siRNA, *ZYX* siRNA: 5′-CUC CUA AGU UUA CUC CUG U-3′, and negative control siRNA: 5′-GGG AAG AUC GGG UUA GAC UUC-3′. Thirty picomoles of siRNA was transfected into HEK293 cell in 24-well plates with Lipofectamin 2000 (Thermo Fisher Scientific) or RNAi MAX (Thermo Fisher Scientific) according to manufacturer’s instruction. 2 days after siRNA transfection, knockdown efficiency of *ZYX* was confirmed by RT-qPCR.

### Yeast two-hybrid assays

The yeast two-hybrid assay was carried out as described previously^42^. Briefly, the yeast AH109 cells (Clontech) was transformed using bait (pGBKT7) and prey (pGADT7) plasmids. pGBKT7 and pGADT7 plasmids encode TRP1 and LEU2 genes, respectively. The transformants were streaked onto SD-WL selection plates (synthetic dextran medium lacking Trp and Leu) and were incubated for 3–5 days. The MAVS vector was constructed by inserting the MAVS full length ORF into the multicloning site of pGBKT7. Yeast two-hybrid screening was performed with human lung cDNA libraries, using SD-WLH and SD-WLHA selection plates, in which Trp/Leu/His and Trp/Leu/His/adenine are removed from synthetic dextran medium, respectively. AH109 genome encodes *HIS3* and *ADE2* reporter genes, which are activated if the two proteins encoded in the prey and bait plasmids physically interact with each other. We obtained several candidate clones, and one candidate encoded a partial ORF of zyxin that encoded the C-terminal region.

### Reporter assay

HEK293 cells (2 × 10^5^ cells/well) were cultured in 24-well plates, and then cells were transfected with empty vector and/or expression vectors as indicated in the figure legends together with p-125luc reporter plasmid (100 ng/well) and an internal control vector, phRL-TK (Promega) (10 ng/well) using Lipofectamine 2000. The p-125luc reporter, in which the human *IFNB1* promoter region (−125 to +1) is fused to luciferase gene, was provided by Dr. T. Taniguchi (University of Tokyo, Tokyo, Japan). The total amount of DNA (500 ng/well) was kept constant by adding empty plasmids.

### Virus preparation and infection

Influenza A virus (PR-8, A/Puerto Rico/8/1934 H1N1) or Poliovirus type 1 Mahoney strain were used for virus assay. Fertilized eggs were used for propagation of influenza A virus, and MDCK cells were used to determine the viral titer. Poliovirus type 1 Mahoney strain was propagated with Vero-derived cells (Vero-SLAM), which was also used to determine viral titer.

### Confocal analysis

HeLa cells were cultured onto a cover glass in a 24-well plate for 1 day, and were transfected with plasmid for the expression of zyxin. The amount of DNA was kept constant by adding empty vector. After 24 h, MitoTracker Red (Thermo Fisher) was treated for 30 min according to manufacturer’s protocols. Cells were fixed with 4% paraformaldehyde in PBS for 30 min in room temperature, and were then permeabilized and blocked with PBS containing 0.2% of Triton X-100 and 5% FBS for 60 min. Permeabilized cells were labeled with anti-HA polyclonal (Sigma Aldrich) Ab in 1% BSA/PBS over night at 4 °C. Cells were washed with PBS and incubated with Alexa Fluor 488-conjugated Ab (Thermo Fisher) in 1% BSA/PBS for 60 min at room temperature. Stained cells were covered with Prolong Diamond Antifade Mountant with DAPI (Thermo Fisher) and observed under a FluoView FV1200 confocal microscope (Olympus).

### Proximity ligation assay (PLA)

HeLa cells cultured on cover glass were transfected with plasmids encoding FLAG-MAVS and Zyxin-HA. Cells were fixed, permeabilized, and labeled with anti-FLAG monoclonal antibody and anti-HA polyclonal antibody in 1% BSA in PBS for 1 hr at room temperature. Subsequently, PLA signals were detected by Duolink *in situ* PLA kit, according to manufacturer’s protocol (Sigma). Briefly, secondary antibodies conjugated with the positive or negative PLA probe were added to the labeled cells. The hybridized probes were then used for visualizing the PLA signals using Duolink detection reagents Green or Red. PLA signals were observed by the confocal microscope (Olympus).

### Immunoprecipitation and immunoblotting

HEK293FT cells were transfected in a 6-well plate with plasmids encoding zyxin, MAVS, RIG-I as indicated in the figures. Cells were lysed with lysis buffer consisting of 20 mM tris-HCl (pH 7.5), 125 mM NaCl, 1 mM EDTA, 10% Glycelol, 1% NP-40, 30 mM NaF, 5 mM Na_3_VO_4_ and protease inhibitor cocktail tablets (Roche). Cell lysates were incubated for 30 min on ice, and were subject to centrifugation at 15,000 rpm for 20 min at 4 °C. A part of supernatants were boiled at 95 °C for 5 min and others were incubated with anti-FLAG antibody at 4 °C for 120 min and then incubation with protein G-Sepharose beads (GE Healthcare) for 4 °C overnight. The beads were washed three times with cold lysis buffer and boiled at 95 °C for 5 min. The proteins were subjected to SDS-PAGE and were transferred onto PVDF membranes (MERCK MILLIPORE). The membrane was blocked with rinse buffer (0.1% tween 20, 10 mM Tris-HCl pH 7.5, 0.8% NaCl, 1 mM EDTA, 5% skim milk) and was incubated with primary antibody at 4 °C overnight, and then was incubated with HRP-conjugated secondary antibody at room temperature at 1 h. The immune complex was visualized with Amersham ECL prime western blotting detection reagent (GE Healthcare) and detected by a ChemiDoc touch (Bio-Rad).

### qPCR

Total RNA was extracted using TRIzol (Invitrogen), according to the manufacturer’s instruction. Reverse transcription reactions of mRNA were performed using the High-Capacity cDNA Reverse Transcription Kit (Life Technologies). qPCR was performed with the Power SYBR Green PCR Master Mix (Life Technologies) using the Step One Real-Time PCR System (Life Technologies). The primer sequences for the *ZYX* gene are following: forward 5′-ATC CTC AGA GGC AGA ATG TGG-3′ and reverse 5′-AAG CAG GCG ATG TGG AAC-3′. The primer sequences for the *IFNB1* gene are following: forward 5′-TGG GAG GTT CTG CAT TAC C-3′ and reverse 5′-CAG CAT CTG CTG GTT GAA-3′. The primer sequences for the IP-10 (*CXCL10*) gene are following: forward 5′-TCC ACG TGT TAG ATC ATT GC-3′ and reverse 5′-GGC CTT CGA TTC TGG ATT CAG-3′. The primer sequences for the *GAPDH* gene were following: forward 5′-CAA TAT GAT TCC ACC CAT GG-3′ and reverse 5′-AAT GAG CCC CAG CTT CTC C-3′. The primer sequences for Influenza A virus gene were following: forward 5′-TGA ACT GAG AAG CAG GTA CTG G-3′ and reverse 5′- GAA TGC TGC CAT AAC GGT TG-3′.

### Statistic analysis

Error bars represent the mean ± standard deviation (SD). Data was analyzed by one-way analysis of variance (ANOVA) and the t-test. Statistic analysis was performed using Prism 7 for Mac OS X software (GraphPad Software).

### Data availability

All data generated or analyzed during this study are included in this published article (and its Supplementary Information failes).

## Electronic supplementary material

Supplemental Information
