# Supplementary material for: Zyxin stabilizes RIG-I and MAVS interactions and promotes type I interferon response
Source: Sci Rep. 2017 Sep 19;7:11905. doi: 10.1038/s41598-017-12224-7 (PMC5605516; doi:10.1038/s41598-017-12224-7)

Supplemental Information

Zyxin stabilizes RIG-I and MAVS interactions and promotes type I interferon response

Kouwaki Takahisa[1], Masaaki Okamoto[1], Hirotake Tsukamoto[1], Yoshimi Fukushima[1], Misako Matsumoto[2], Tsukasa Seya[2], and *Hiroyuki Oshiumi[1,3]

[1]Department of Immunology, Graduate School of Medical Sciences, Faculty of Life Sciences, Kumamoto University, 1-1-1, Honjo, Chuo-ku, Kumamoto 860-8556 Japan
[2]Department of Microbiology and Immunology, Graduate School of Medicine, Hokkaido University, Kita-Ku Sapporo 060-8556 Japan
[3]JST, PRESTO, 1-1-1- Honjo, Chuo-ku, Kumamoto 060-8556 Japan

Figure legends

Supplemental Figure S1. Zyxin expression patterns in human cells

(a–c) HeLa, THP-1 macrophages, and A549 cells were stimulated with polyI:C (a–c) or IFN-α (d, e). Total RNA was extracted at indicated time points, and the expression of *MAVS* (a), *ZYX* (b, d), *IFNB1* (c), and *CXCL10* (e) were determined by RT-qPCR and were normalized to *GAPDH*.

(f) HeLa cells were stimulated with polyI:C transfection, and cell lysates were prepared at indicated time points after stimulation. The protein expression levels of phosphorylated-zyxin (p-zyxin) and total zyxin were determined by western blotting with anti-p-zyxin and anti-zyxin antibodies.

Supplemental Figure S2. Full blot images

(a) For Fig. 1b. (b) For Fig. 1c. Asterisk (*) represents a degradation product of zyxin. (c) For Fig. 1d. (d) Fig. 1e. (e) For Fig. 3c. (f) For Fig. 4i. (g) For Fig. 4j. (h) For Fig. 4k.

Supplemental Figure S3. Full blot images

(a) For Fig. 5a. (b) For Fig. 5b. (c) For Fig. 5d. (d) For Fig. 5e. (e) For Fig. 5f. (f) For Fig. 6a. Asterisk (*) represents a short form of MAVS caused by alternative splicing. (g) For Fig. 6b. Asterisk (*) represents a short form of MAVS caused by alternative splicing.

Supplemental Figure S1

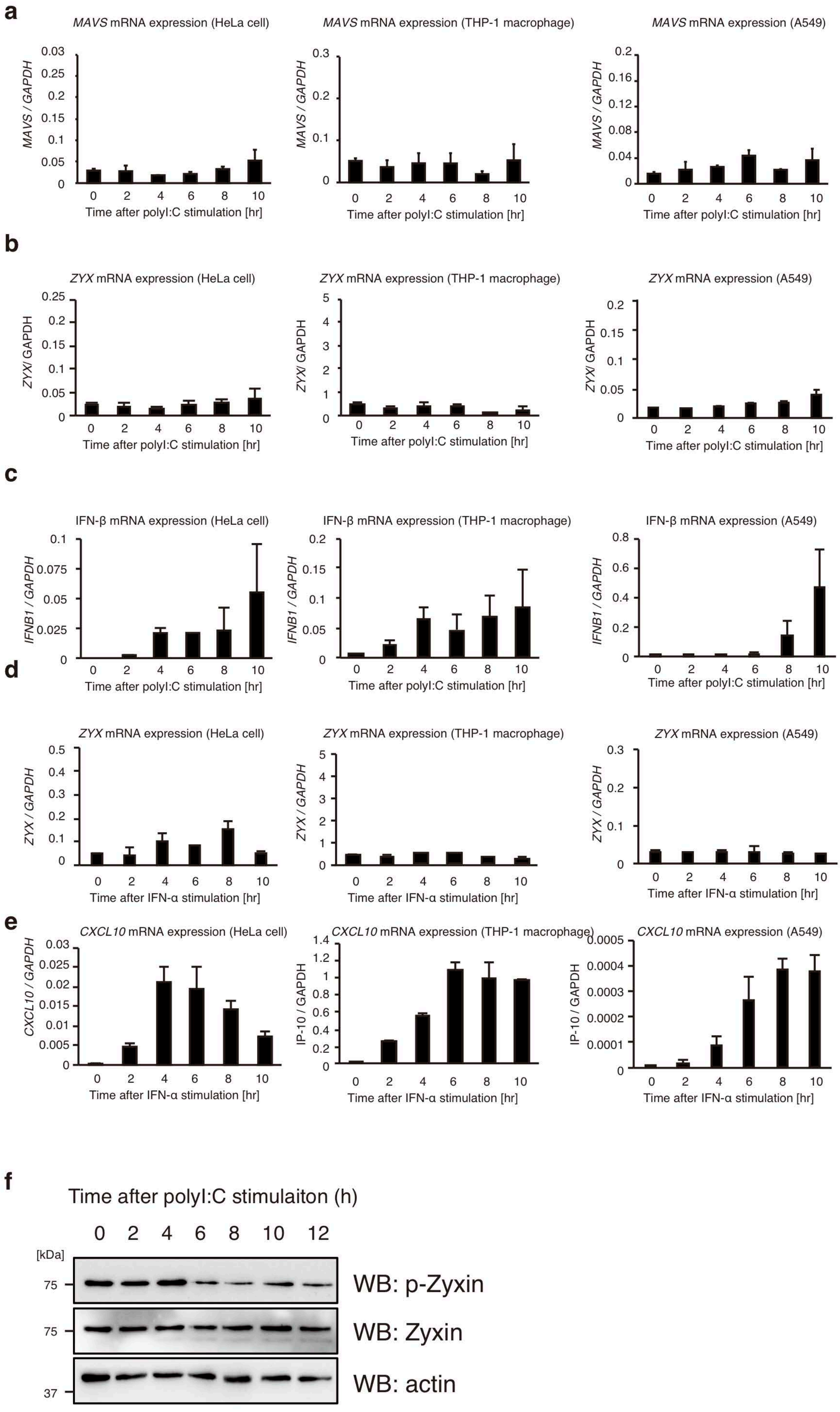

Supplemental Figure S2

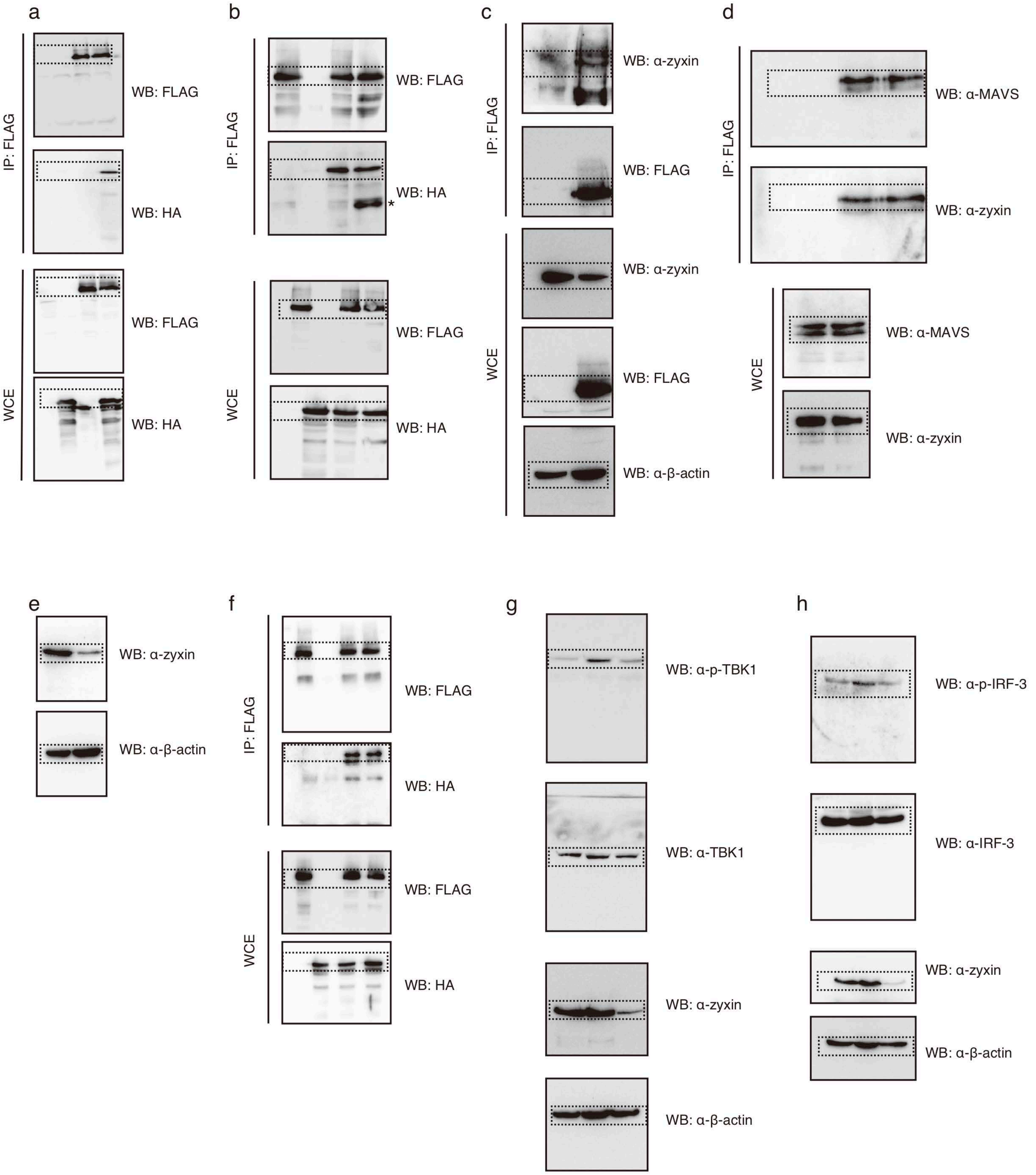

Supplemental Figure S3

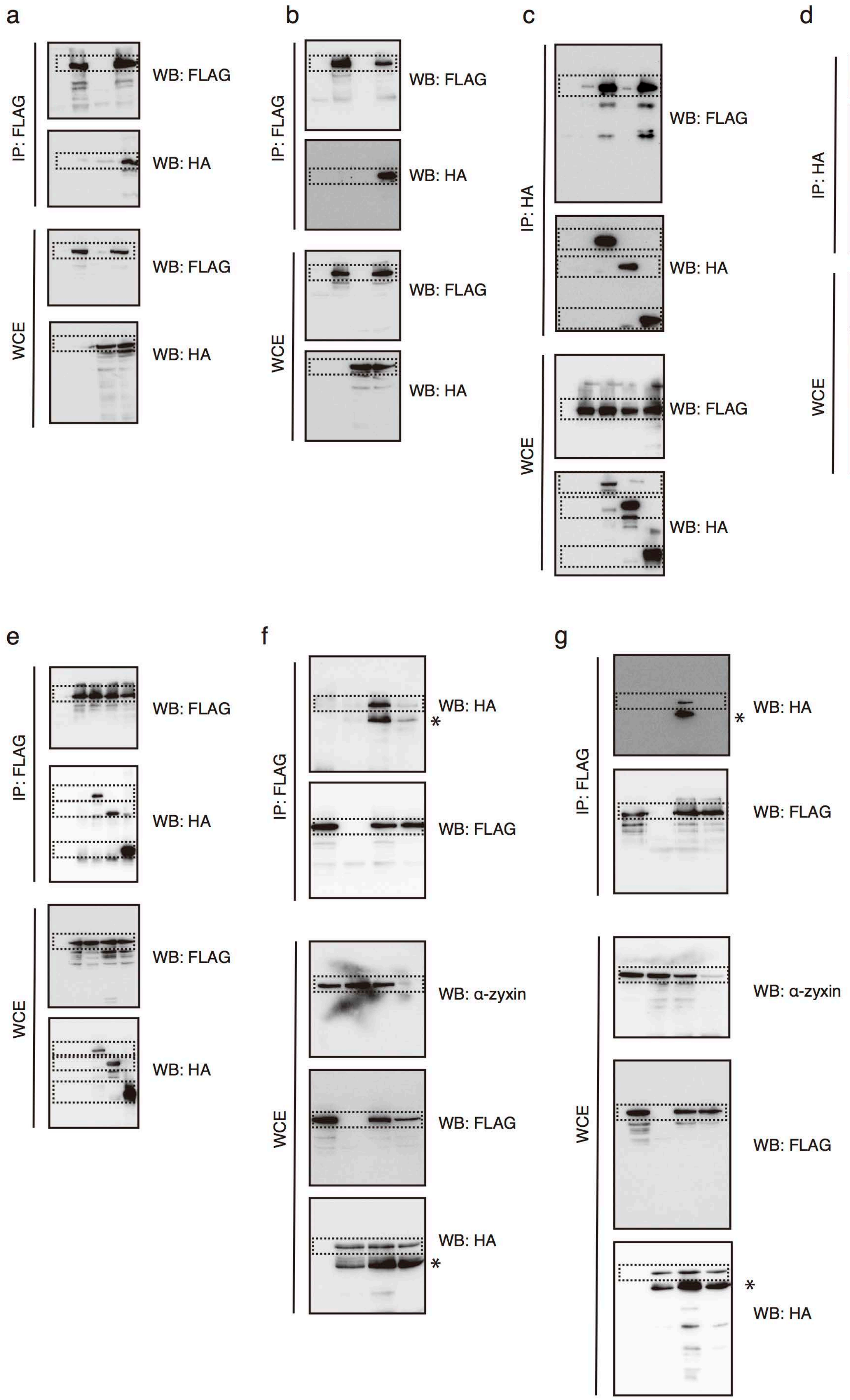

Supplement: Supplementary file 1 — Supplemental Information [file 41598_2017_12224_MOESM1_ESM.pdf]
